# Banxia Shumi Decoction Alleviates PCPA‐Induced Insomnia and Regulates ADAM10 Expression via Melatonin Signaling in Rats

**DOI:** 10.1002/cns.70988

**Published:** 2026-09-20

**Authors:** Baojun Guo, Yuqin Tang, Yongqiang Zhang, Yeke Wu, Ranran Gao

**Affiliations:** ^1^ Department of TCM Henan Provincial People's Hospital, Zhengzhou University Zhengzhou Henan China; ^2^ Clinical Bioinformatics Experimental Center Henan Provincial People's Hospital, Zhengzhou University Zhengzhou Henan China; ^3^ Henan Academy of Innovations in Medical Science Henan Academy of Medical Sciences Zhengzhou Henan China; ^4^ Department of Stomatology Hospital of Chengdu University of Traditional Chinese Medicine Chengdu Sichuan China

**Keywords:** ADAM10, Banxia Shumi decoction, insomnia, melatonin, network pharmacology

## Abstract

**Background:**

Insomnia is a pathological sleep disorder marked by persistent sleep‐onset difficulties. This study explored the therapeutic effects and underlying mechanism of Banxia Shumi Decoction (BXSMD) in treating insomnia.

**Methods:**

This study used network pharmacology to predict the core targets and signaling pathways of BXSMD in insomnia intervention. A rat insomnia model was constructed via 4‐Chloro‐DL‐phenylalanine (PCPA) to assess BXSMD's behavioral impacts. ELISA, qRT‐PCR, Western blot, and IHC were applied to detect the expression of 5‐hydroxytryptamine (5‐HT), melatonin (MT), MT1/MT2 receptors, and related factors (CLOCK, PER1, CaMKII, p‐CREB/CREB, GABARAP, mGLuR1, ADAM10) in the rat suprachiasmatic nucleus (SCN), hippocampus, and serum. The non‐selective melatonin receptor antagonist luzindole was administered to explore its influence on BXSMD‐regulated expression of the above molecules.

**Results:**

Compared with the model group, the BXSMD group showed shortened sleep latency and prolonged sleep duration. BXSMD significantly elevated 5‐HT, MT, and MT1/MT2 levels. BXSMD upregulated the mRNA and protein expression of downstream effector molecules including CLOCK, PER1, CaMKII, and GABARAP, downregulated mGLuR1 expression, and increased the expression of ADAM10. Notably, CREB mRNA expression remained unchanged, while BXSMD significantly increased the p‐CREB/CREB protein ratio. The regulatory effects of BXSMD on all altered molecules were significantly reversed by luzindole.

**Conclusion:**

BXSMD effectively alleviates PCPA‐induced insomnia in rats. The mechanism relies on MT1/MT2 as core targets, which mediate the regulation of sleep‐related molecules (CLOCK, PER1, CaMKII, p‐CREB/CREB, GABARAP, and mGluR1), as well as the membrane protein shedding‐related molecule ADAM10.

## Graphical abstract

This study explored BXSMD's effect and mechanism on rat insomnia. Pentobarbital‐induced sleep tests showed that BXSMD shortened sleep latency and prolonged sleep duration compared with the model group. The mechanism relies on MT1/MT2 as core targets, which mediate the regulation of sleep‐related molecules (CLOCK, PER1, CaMKⅡ, p‐CREB/CREB, GABARAP, and mGluR1), as well as membrane protein shedding‐related molecule ADAM10.

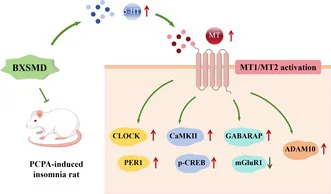

**Abbreviations**
5‐HT: 5‐hydroxytryptamine
BXSMD: Banxia Shumi Decoction
CaMKII: Calmodulin kinase II
CREB: cAMP response element binding protein
DIA: diazepam
ELISA: enzyme‐linked immunosorbent assay
GABARAP: gamma‐aminobutyric acid type A receptor‐associated protein
Glu: glutamate
GO: Gene Ontology
IHC: Immunohistochemistry
KEGG: Kyoto Encyclopedia of Genes and Genomes
MT: melatonin
MT1: melatonin receptor type 1
MT2: melatonin receptor type 2
MTR: melatonin receptor
OF: open field
PCPA: 4‐Chloro‐DL‐phenylalanine
PPI: protein–protein interaction
qRT‐PCR: Quantitative real time RT‐PCR
SCN: suprachiasmatic nuclei
SD: Sprague Dawley
SL: sleep latency
ST: sleep time
TC: thalamocortical
WB: Western blotting

## 1 Introduction

Insomnia is a pathological sleep disorder characterized by persistent and frequent difficulties in falling asleep, resulting in chronic sleep dissatisfaction [1, 2]. According to the World Health Organization, insomnia is the most common type of clinical sleep disorder. Approximately 27% of the world's population suffers from insomnia, which has a serious negative impact on people's normal life and work [3]. The prevalence of insomnia varies considerably across different studies, ranging from 4% to 36% among adolescents and 9% to 50% among adults [4]. Mental and physical disorders, including depression, anxiety, hypertension, diabetes, and cardiovascular disease, are caused by persistent insomnia [5, 6].

The hippocampus plays an important role in the regulation of sleep and promotes slow‐wave sleep [7]. The suprachiasmatic nuclei (SCN), located in the hypothalamus above the optic chiasm, make up the human pacemaker known as the circadian or biological clock [8]. MT is known as the sleep hormone. In humans, circulating melatonin levels begin to rise in the evening, peak at midnight, and then drop to their lowest levels by the end of the night. After being synthesized in the pineal gland, MT is released into the cerebrospinal fluid through the pineal sulcus. It may reach all brain regions and the circulatory system, subsequently entering the periphery [9]. MT can cross the blood–brain barrier and bind to the corresponding melatonin receptor (MTR) subtypes, which are MT1, MT2, and MT3, MT1 and MT2 have been found to be associated with sleep. MT regulates sleep and restores circadian rhythms by interacting with MT1 and MT2 [10]. Per1 and CLOCK are two of the most important biological clock genes/proteins involved in the transcription‐translation loop and control core circadian rhythms [11]. Oscillatory rhythms regulated by Per1 and CLOCK have been reported to be severely disrupted after MT1 knockdown. MT2 primarily regulates non‐rapid eye movement sleep, and there is evidence that MT2 activated by MT promotes an increase in intracellular calcium ions as well as the expression of Calmodulin kinase II (CaMKII), which enhances the phosphorylation level of cAMP response element binding protein (CREB) [12]. However, whether BXSMD regulates circadian rhythm‐related molecules (CLOCK, PER1) and calcium signaling‐related molecules (CaMKII, p‐CREB) via MT1/MT2 remains unclear.

Neuronal excitability is homeostatically modulated by the inhibitory neurotransmitter gamma‐aminobutyric acid (GABA) and the excitatory neurotransmitter glutamate (Glu), both of which are essential for normal sleep architecture [13]. GABA is synthesized from Glu by glutamic acid decarboxylase and plays a key role in preventing insomnia [14]. Glu levels are significantly elevated in PCPA‐induced insomnia rats [15]. GABA(A) receptor‐associated protein (GABARAP) participates in the intracellular trafficking and membrane localization of GABA(A) receptors, which are essential for maintaining normal GABAergic inhibitory neurotransmission [16]. The metabotropic glutamate receptor mGluR1 is highly expressed in thalamocortical neurons; its activation enhances neuronal excitability and is closely related to sleep–wake regulation and hippocampal synaptic plasticity [17, 18]. However, whether BXSMD regulates GABARAP and mGluR1 expression through the MT1/MT2 pathway remains unclear.

ADAM10 is a type I transmembrane protein that acts as a key α‐secretase. As a multifunctional sheddase, ADAM10 cleaves more than 40 membrane proteins, including APP, Notch, TNF‐α precursor, and ErbB receptors, which are widely involved in brain pathology, inflammation, and cancer [19]. Melatonin has been reported to significantly enhance the transcription of ADAM10 through CREB and Oct‐1 [20]. Moreover, MT can regulate ADAM10 protein activity through MT1/MT2 receptors, protect neurons and glial cells from Aβ‐induced neurotoxicity and oxidative stress, and delay neurodegeneration, thereby improving Alzheimer's disease [19]. However, whether BXSMD improves insomnia by regulating ADAM10 expression in a MT1/MT2‐dependent manner has not been explored.

A variety of sedative‐hypnotic drugs, including benzodiazepines, antihistamines, and antidepressants, are mostly used clinically. Although these medications offer short‐term relief, they are often accompanied by side effects such as dizziness, drowsiness, and drug dependence, making them unsuitable for long‐term use [21, 22]. In order to reduce the adverse effects in treated insomnia patients, more and more scholars' research focuses on natural medicines. BXSMD is a well‐known Chinese formula used to treat insomnia in China [23]. BXSMD is a classic formula composed of two medicines: *Rhizoma Pinelliae Praeparatum* (Banxia) and *Sorghum vulgare* (Shumi). Modern pharmacological studies have found that the main components of Banxia include flavonoids, fatty acids, organic acids, etc., which have anti‐inflammatory, antioxidant, antidepressant, and sedative‐hypnotic effects. BXSMD has a good sedative‐hypnotic effect on insomniac rats with respect to the circadian clock gene [23, 24].

Our preliminary studies have demonstrated that BXSMD enhances MT expression by activating the hippocampal BDNF/TrkB/CREB pathway, thereby improving sleep disturbances and neural damage in male rats with insomnia [25]. On this basis, we further take MT1/MT2 as core necessary upstream targets, and systematically investigate their regulatory effects on sleep‐related molecules (CLOCK, PER1, CaMKII, p‐CREB/CREB, GABARAP, and mGluR1), as well as the membrane protein shedding‐related molecule ADAM10 in PCPA‐induced insomniac rats. This study aims to clarify the key regulatory nodes in the signaling axis of BXSMD, so as to provide a more rigorous scientific basis for its mechanism and application in the intervention of insomnia.

## 2 Materials and Methods

### 2.1 Animals and Insomniac Model With PCPA

Eighty Sprague–Dawley (SD) male rats of SPF grade (aged 8 weeks, weight 200–250 g) were purchased from Spiff Biotechnology (Beijing, China). The animals were housed under standard SPF conditions in a controlled environment maintained at a constant temperature (21°C–26°C) and humidity (40%–70%), with a 12‐h light/dark cycle. Food and water were provided ad libitum.

Insomnia can be induced in rats through the intraperitoneal injection of 4‐Chloro‐DL‐phenylalanine (PCPA) [26]. The rats received intraperitoneal injections of PCPA (S27337, Yuanye Bio‐Technology, Shanghai, China) at a dosage of 350 mg/kg daily. After 3 days of PCPA injection, the rats had altered circadian rhythms and demonstrated continuous awake throughout the day, demonstrating the effective creation of the insomnia model. Diazepam was administered at 10 mg/kg/d by gavage. Luzindole (L2407, Sigma, St. Louis, MO, USA) was administered at 10 mg/kg/d by intraperitoneal injection. DMSO (HY‐Y0320C, MCE, Shanghai, China) was administered via intraperitoneal injection, with the same volume as Luzindole.

After 14 consecutive days of BXSMD administration, male rats from each group were subjected to a series of behavioral tests in the following sequence: the spontaneous activity test, open field test, Morris water maze test, and pentobarbital‐induced sleep test. Upon completion of all behavioral assessments, the body weights of all rats were measured and recorded. Finally, the rats were euthanized under anesthesia, and brain tissues along with blood samples were collected for subsequent molecular and histopathological analyses.

### 2.2 Preparation of BXSMD

BXSMD first comprises only two medicines: Banxia and Shumi. Banxia is provided by Chongqing Guochengtang Pharmaceutical. Shumi is purchased from Bozhou Huaxin Traditional Chinese Medicine Decoction Technology.

The various Chinese herbal medicines in BXSMD are decocted and concentrated, then administered via gastric lavage. The model and blank control groups were administered by gastric infusion with saline solution. The high, middle, and low groups were treated by 4.69, 9.38, and 18.75 g/kg, once a day, for 14 consecutive days.

### 2.3 Metabolomics and Network Pharmacology Research

The relevant targets of the main active ingredients of BXSMD were obtained from the TCSMP database. After screening the active ingredients, the Swiss Target prediction database was utilized for ingredient target prediction. Insomnia‐related genes were obtained using the Gene Cards database. The intersection of the main targets of BXSMD and insomnia‐related genes was taken to make a Venn diagram. The intersection targets were imported into the STRING database, and the protein–protein interaction (PPI) network was constructed by removing the unrelated nodes. The two intersecting targets were subjected to Gene Ontology (GO) biological process analysis and Kyoto Encyclopedia of Genes and Genomes (KEGG) signaling pathway enrichment analysis.

### 2.4 Autonomous Activity Experiment

This experiment was conducted using an autonomous activity video analysis system. During testing, rats were exposed to light from 8:00 to 20:00 daily and darkness from 20:01 to 7:59. After a 3‐min acclimation period, testing commenced. Every 3 h, the distance and duration of autonomous activity within a 2‐min window were recorded to analyze circadian activity patterns.

### 2.5 Open Field Test

Experimental animals were transported in advance to the behavioral laboratory's dedicated temporary cage racks. After approximately 3 h of environmental acclimation, rats were placed at the center of the open field (OF) apparatus. The OF lid was closed swiftly and gently. The video recording system was activated to document rat activity within the OF for a total duration of 5 min. Observation metrics comprised horizontal activity scores (number of grid squares traversed) and vertical activity scores (number of hindlimb rearing episodes).

### 2.6 Morris Water Maze Evaluation

The experiment began by allowing rats to freely swim in the water tank for 2 min to familiarize them with the environment. The entire experiment lasted 5 days, with training sessions conducted at fixed times each day, totaling four trials per time slot. At the start of training, the platform was placed in the NW quadrant. Rats were placed into the water facing the wall from any of the four starting points along the tank wall. A video recording system captured the time taken to locate the platform (escape latency) and swimming trajectories. Rats were placed in the water from four distinct starting points (different quadrants) during each training session. In the Morris water maze procedure, if a rat located the platform or failed to find it within 120 s (latency recorded as 120 s), the experimenter retrieved the rat onto the platform. The rat rested on the platform for 15 s before the next trial commenced. The daily learning performance was calculated as the average of the rat's four training sessions. On Day 6, the original platform was removed. Rats were placed in the water at a randomly selected entry point (all rats must use the same entry point), and the number of times the rat crossed the original platform within 2 min was recorded.

### 2.7 Pentobarbital‐Induced Sleep Test

Twelve hours after the second intraperitoneal injection of PCPA, the pentobarbital sodium test was conducted. Rats were sequentially administered intraperitoneal injections at a concentration of 50 mg/mL and a dose of 35 mg/kg. The rats were then placed supine on the testing platform. The time interval from sodium pentobarbital injection to the disappearance of the righting reflex was observed and recorded as sleep latency (SL). The time interval from the disappearance of the righting reflex to its recovery was recorded as sleep time (ST).

### 2.8 Enzyme‐Linked Immunosorbent Assay (ELISA)

The expression of 5‐HT (E‐EL‐0033c, Elabscience, Wuhan, China), MT (E‐EL‐R0031c, Elabscience), MT1 (ABIN6963194, antibodies‐online, Aachen, Germany), MT2 (ABIN6957777, antibodies‐online) in hypothalamic supraoptic nucleus, hippocampus, and serum were measured by ELISA kits. The experiments were performed according to the instructions.

### 2.9 Quantitative Real Time RT‐PCR (qRT‐PCR)

Total RNA was isolated using Trizol Invitrogen (15,596,018, Invitrogen, Carlsbad, CA, USA) according to the manufacturer's instructions and was reverse transcribed by using PrimeScript RT reagent Kit (RR037Q, Takara, Tokyo, Japan). Quantitative real‐time PCR analysis was performed under the following conditions: 30 s at 95°C followed by 40 cycles at 95°C for 3 s, and 60°C for 30 s using Real time PCR system (ABI 7900HT, Applied Biosystems, Foster City, CA, USA). For quantification of gene expression, the 2^−ΔΔCt^ method was used. GAPDH expression was used for normalization. Primers used in this article are listed in Table 1.

**TABLE 1:** Primers used in qRT‐PCR.

Gene	Forward sequence (5′‐3′)	Reverse sequence (5′‐3′)
ADAM10 Rat	GGCTGGGAGGTCAGTATGGAA	CTCGTGTGAGACTGCTCGTT
CLOCK Rat	CTTACAGACATCTCGGTTGCTCC	TGGTGCGAAGGAGGGAAAGT
MT1 Rat	TGAGTGTCATTGGCTCGGTA	TCAGGAACACGTAGCACAGG
MT2 Rat	AGACAGCCAGCACCCAATAC	TTCTCTCAGCCTTTGCCTTC
Per1 Rat	CGAAGTTTGAGCTCCCGAAGT	CCAGGCCCGGAGAACCTTTTT
CAMKII Rat	CACCTTTCTGGGATGAGGAC	GTTTTTGGCTTCAGGGGTAA
CREB Rat	CAGACAACCAGCAGAGTGGA	TACAGTGGGAGCAGATGACG
GAD65 Rat	AGCTGGAACCACTGTGTACG	ACCAGGAGAGCCGAACATTG
GABARAP Rat	AAAAGCTCCCAAAGCTCGGA	ACTTCACAGACCGTAGACGC
GAPDH	GCACCGTCAAGGCTGAGAAC	TGGTGAAGACGCCAGTGGA

### 2.10 Western Blotting

Hippocampal tissues or hypothalamic tissues were washed with pre‐cooled PBS, then lysed on ice using RIPA Lysis Buffer (BL504A, Bio sharp, Hefei, China) with a Protease Inhibitor (A32955, Thermo fisher, Waltham, MA, USA) for 30 min and centrifuged at 12,000 g at 4°C for 15 min. The supernatant was collected and its protein concentration was measured with the Enhanced BCA Protein Assay Kit (BL521A, Bio sharp). After denaturation, all the proteins were separated by different SDS‐PAGE and transferred to PVDF membranes. The PVDF membranes were then blocked with 5% skim milk for 2 h at room temperature and incubated with primary antibodies CREB (ab31387, Abcam, Cambridge, MA, USA), p‐CREB (ab194687, Abcam), CLOCK (ab308571, Abcam), Per1 (ab58886, Abcam), GABARAP (BM5326, BOSTER, Wuhan, China), CaMKII (ab52476, Abcam), mGLuR1 (12,551, CST, Danvers, MA, USA), ADAM10 (ab124695, Abcam), and GAPDH (60004‐1‐Ig, Protein tech, Wuhan, China) overnight at 4°C. Then, the membranes were washed three times with TBST and then incubated with the appropriate horseradish peroxidase‐labeled secondary antibodies for 60 min at room temperature. An enhanced chemiluminescence reagent was used to view the reaction. Standardization was performed using GAPDH.

### 2.11 Immunohistochemistry (IHC)

Hippocampal and hypothalamic region samples were fixed in 4% paraformaldehyde, dehydrated, and then embedded in paraffin. Cut the embedded tissue into 4 μm paraffin sections. Place the sections in a 37°C water bath to unroll, mount, and dry them. Following deparaffinization, rehydration, and antigen retrieval, tissue sections were incubated with an appropriate amount of endogenous peroxidase blocking agent at room temperature for 10 min and washed with PBS. Subsequently, the sections were incubated with a primary antibody against ADAM10 (PA5‐81301, Invitrogen) at 37°C for 30 min. After washing, enzyme‐labeled goat anti‐rat/rabbit IgG polymer was applied and incubated at 37°C for 20 min, followed by another washing step. DAB (G1212‐200 T, servicebio, Wuhan, China) was then added for color development, and the sections were counterstained with hematoxylin for 2 min, differentiated with differentiation solution for 15 s, and blued with bluing solution for 1 min, with washing steps between each procedure. Finally, the sections were dehydrated, cleared, mounted, imaged, and observed under a microscope (Leica, Germany).

### 2.12 Statistical Analysis

Each assay was performed for three times. Data were analyzed by GraphPad Prism 8.0 (La Jolla, CA, USA) and expressed as mean ± standard deviation. One‐way ANOVA test was used for multiple variable comparison. *p* < 0.05 was considered a significant difference.

## 3 Results

### 3.1 Network Pharmacology Forecasted the Potential Mechanism of BXSMD on Insomnia

**FIGURE 1:**
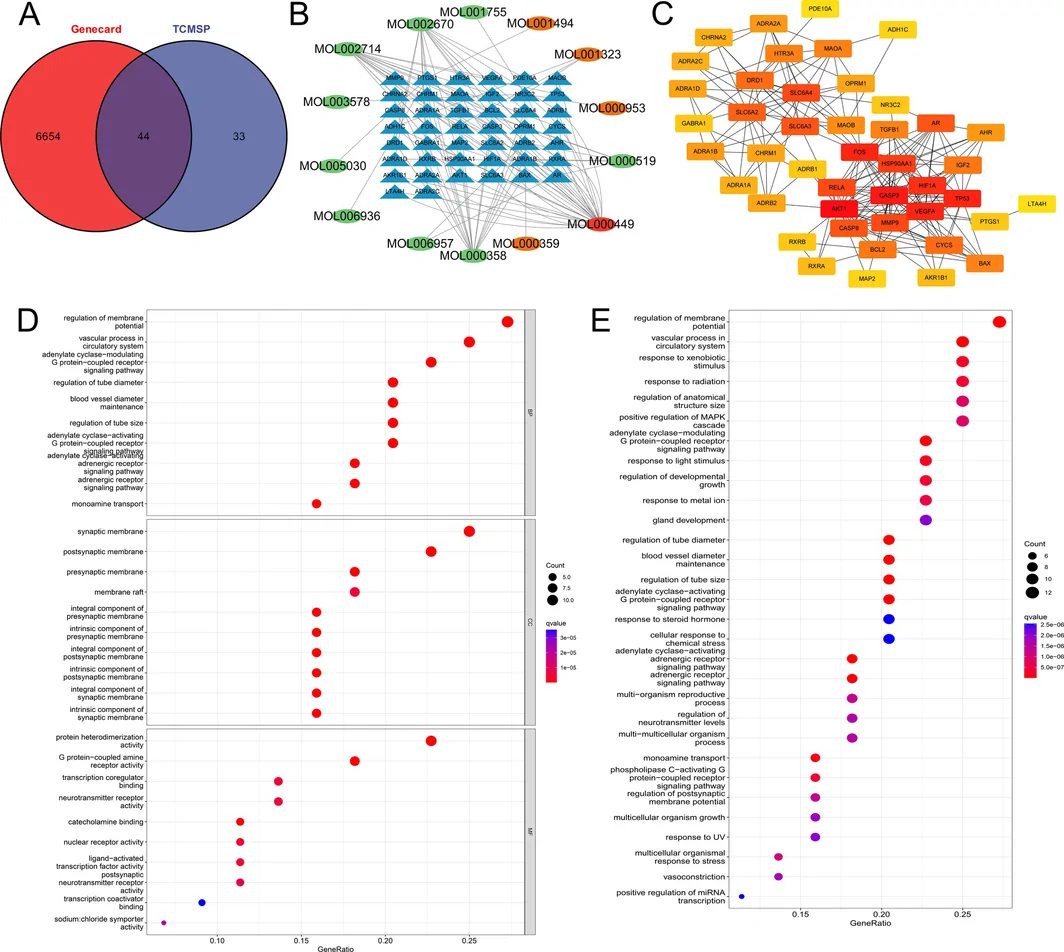
Network pharmacology‐based prediction of the potential therapeutic mechanisms of BXSMD in treating insomnia. (A) Venn diagram of BXSMD and potential insomnia target genes. (B) Activity‐target network diagram of intersecting genes. (C) Visualization of PPI protein interaction network. (D) GO functional enrichment analysis. (E) KEGG pathway enrichment analysis.

We explored the effects of BXSMD on insomnia using network pharmacology methods. A total of 77 potential targets of BXSMD components were obtained from TCMSP, while 6698 insomnia‐associated genes were collated from GeneCards. Intersection of these datasets yielded 44 overlapping genes, which were defined as potential targets for BXSMD's anti‐insomnia action (Figure 1A). A compound‐target network was then generated from these 44 genes using Cytoscape, visualizing the interactions between 14 bioactive ingredients and their respective targets (Figure 1B). Further PPI network analysis highlighted the top 7 hub targets based on node degree (Figure 1C). GO and KEGG enrichment analyses demonstrated significant enrichment in key processes including “regulation of membrane potential,”, “vascular process in circulatory system,” and “adenylate cyclase‐modulating G‐protein coupled receptor signaling pathway” (Figure 1D,E). Based on the overlapping genes between BXSMD and insomnia‐related genes, we hypothesize that BXSMD may have therapeutic effects on insomnia.

### 3.2 BXSMD Demonstrates Therapeutic Effects on Rat Insomnia

**FIGURE 2:**
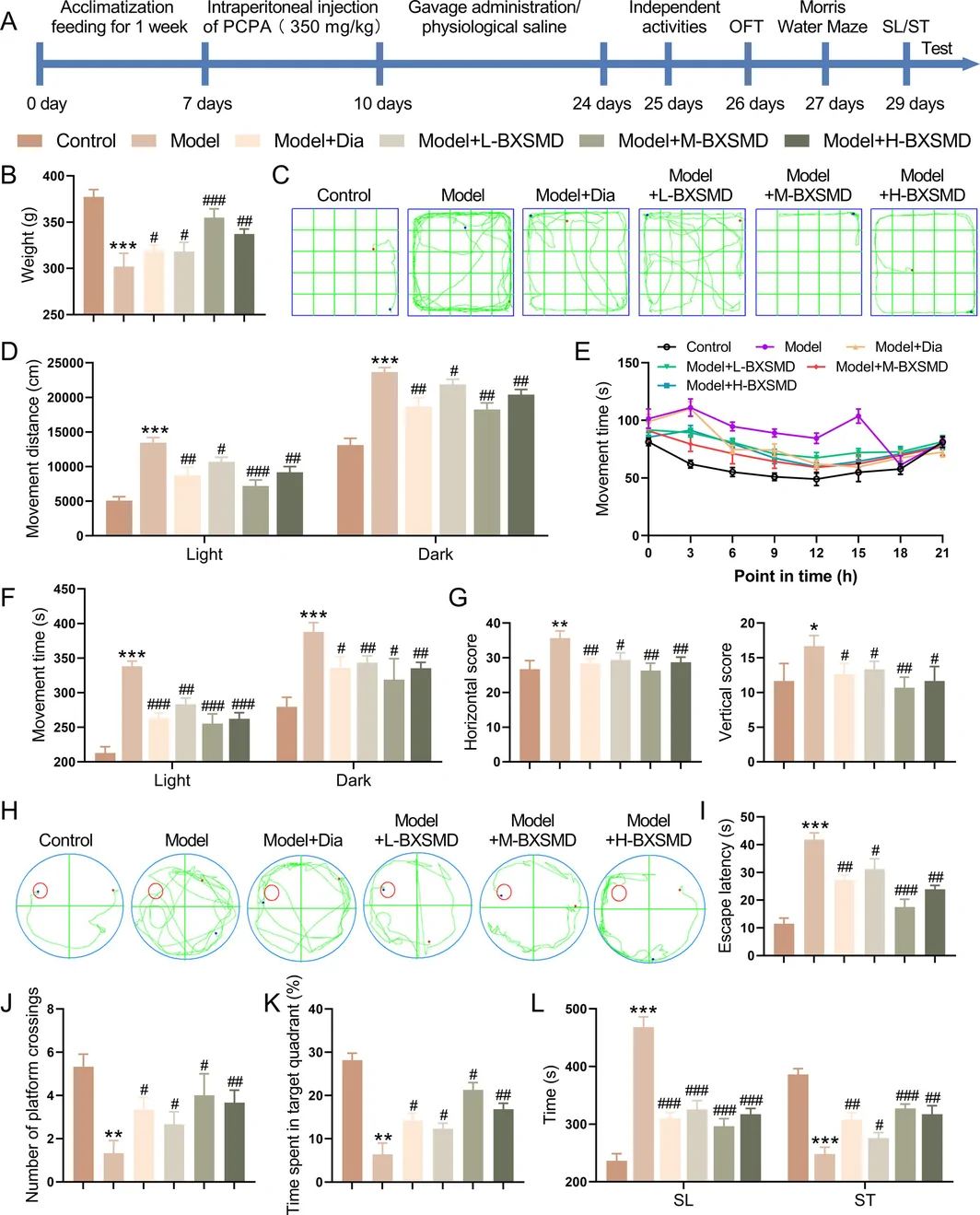
BXSMD demonstrates therapeutic effects on insomnia in rats. (A) Experimental timeline of BXSMD treatment in the insomnia rats. (B) Body weights of rats in each group (F = 52.39; *p* < 0.0001). (C) Representative movement trajectories of rats from different groups within 2 min. (D) Total distance traveled by rats during spontaneous activity (F_(Light)_ = 36.34, *p* < 0.0001; F_(Dark)_ = 46.77, *p* < 0.0001). (E) Spontaneous activity duration of rats at different time points. (F) Total spontaneous activity duration for rats in each group (F_(Light)_ = 53.85, *p* < 0.0001; F_(Dark)_ = 12.99, *p* = 0.0002). (G) Scores for horizontal and vertical movement in the open field test (F_(horizontal)_ = 8.625, *p* = 0.0011; F_(vertical)_ = 4.239, *p* = 0.0188). (H–K) The Morris water maze experiment evaluated the swimming trajectories, escape latency, number of platform crossings, and percentage of time spent in the target quadrant for rats in each group (F_(I)_ = 54.09, *p* < 0.0001; F_(J)_ = 12.13, *p* = 0.0002; F_(K)_ = 54.02, *p* < 0.0001). (L) Pentobarbital‐induced sleep test used to detect SL and ST in insomniac rats (F_(SL)_ = 98.32, *p* < 0.0001; F_(ST)_ = 52.14, *p* < 0.0001). *p < 0.05, **p < 0.01, ***p < 0.001 vs. Control; ^#^ *p* < 0.05, ^##^ *p* < 0.01, ^###^ *p* < 0.001 vs. Model.

To investigate the therapeutic effects of BXSMD on insomnia, we first established a rat model of insomnia using PCPA (Figure 2A). The results showed that rats in the model group exhibited a significant reduction in body weight, while treatment with diazepam and various doses of BXSMD led to a significant recovery in body weight (Figure 2B). We further evaluated the effects of BXSMD on spontaneous locomotor activity in insomnia model rats. As shown in Figure 2C, the movement trajectories of rats in the model group were significantly increased, whereas all drug‐treated groups showed markedly reduced trajectories. Consistently, both movement distance and duration were significantly increased in the model group but were significantly decreased after treatment with diazepam or BXSMD (Figure 2D–F). In the open field test, compared with the model group, all treatment groups exhibited reduced horizontal and vertical movement scores (Figure 2G). In the Morris water maze test, the model group displayed more complex swimming trajectories, which were simplified in the diazepam‐ and BXSMD‐treated groups (Figure 2H). Escape latency was significantly prolonged in the model group but was significantly shortened following treatment with diazepam or BXSMD (Figure 2I). Additionally, the number of platform crossings and the time spent in the target quadrant were significantly reduced in the model group compared with the control group, and these deficits were reversed by drug treatments (Figure 2J,K). Finally, in the pentobarbital‐induced hypnosis test, the model group showed significantly prolonged sleep latency (SL) and shortened sleep time (ST), both of which were reversed by diazepam and different doses of BXSMD (Figure 2L). Collectively, these results demonstrate that BXSMD significantly ameliorates insomnia‐like behaviors in rats.

### 3.3 BXSMD Increased 5‐HT and MT Signaling Expression in Insomniac Rats

**FIGURE 3:**
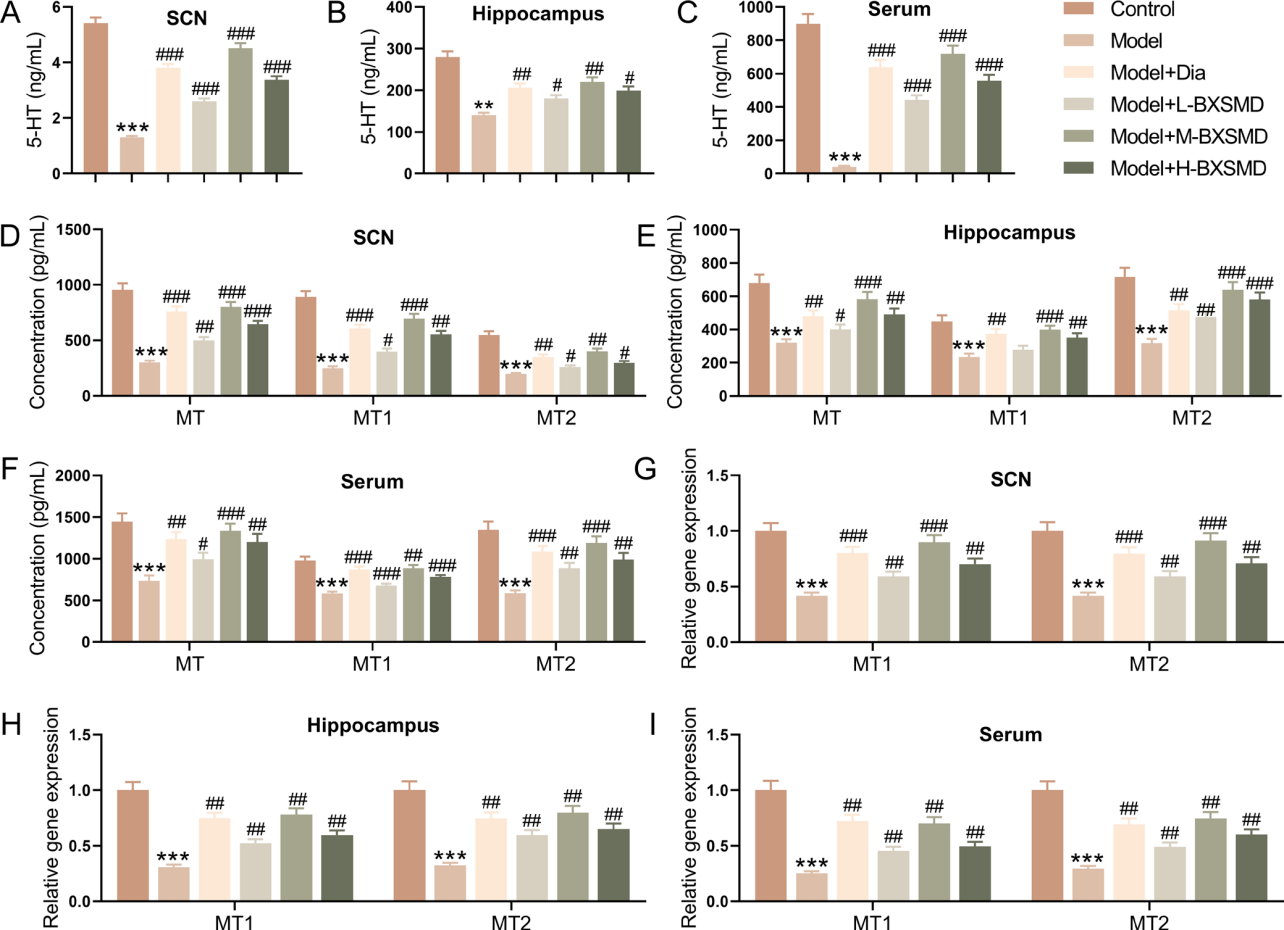
BXSMD increased the levels of 5‐HT, MT, and MT receptors in insomniac rats. (A–C) ELISA was performed to detect the 5‐HT content in the SCN, hippocampus, and serum (F _(A)_ = 301.1, *p* < 0.0001; F _(B)_ = 59.00, *p* < 0.0001; F _(C)_ = 152.1, *p* < 0.0001). (D–F) ELISA was employed to measure the MT, MT1, and MT2 levels in the SCN, hippocampus, and serum (D: F_(MT)_ = 95.81, *p* < 0.0001; F_(MT1)_ = 125.7, *p* < 0.0001; F_(MT2)_ = 89.09, *p* < 0.0001; E: F_(MT)_ = 35.79, *p* < 0.0001; F_(MT1)_ = 25.91, *p* < 0.0001; F_(MT2)_ = 34.63, *p* < 0.0001; F: F_(MT)_ = 26.71, *p* < 0.0001; F_(MT1)_ = 56.31, *p* < 0.0001; F_(MT2)_ = 38.86, *p* < 0.0001). (G–I) qRT‐PCR was applied to quantify the MT, MT1, and MT2 mRNA expression in the SCN, hippocampus, and serum (G: F_(MT1)_ = 47.20, *p* < 0.0001; F_(MT2)_ = 42.00, *p* < 0.0001; H: F_(MT1)_ = 72.36, *p* < 0.0001; F_(MT2)_ = 51.72, *p* < 0.0001; I: F_(MT1)_ = 74.55, *p* < 0.0001; F_(MT2)_ = 62.47, *p* < 0.0001). **p < 0.01, ***p < 0.001 vs. Control; ^#^ *p* < 0.05, ^##^ *p* < 0.01, ^###^ *p* < 0.001 vs. Model.

MT is synthetically derived from 5‐HT [27]. To elucidate the effects of BXSMD on MT in insomnia rats, we measured the levels of 5‐HT, MT, and MT receptors MT1 and MT2. Through ELISA testing, we quantified the content of 5‐HT, MT, MT1, and MT2 in the SCN, hippocampus, and serum of rats from each group (Figure 3A–F). The model group exhibited significantly decreased levels of 5‐HT, MT, MT1, and MT2. Treatment with diazepam and BXSMD significantly increased the levels of 5‐HT, MT, MT1, and MT2. Consistent with these findings, qRT‐PCR analysis of MT1 and MT2 gene expression in the SCN, hippocampus, and serum showed similar trends (Figure 3G–I). These results collectively indicate that BXSMD upregulates the expression of 5‐HT, MT, MT1, and MT2 in insomnia rats. Moreover, M‐BXSMD demonstrated the optimal therapeutic effect, so this dosage was used in all subsequent experiments.

### 3.4 BXSMD Regulated the Expression of Sleep‐Related Molecules in Insomniac Rats

**FIGURE 4:**
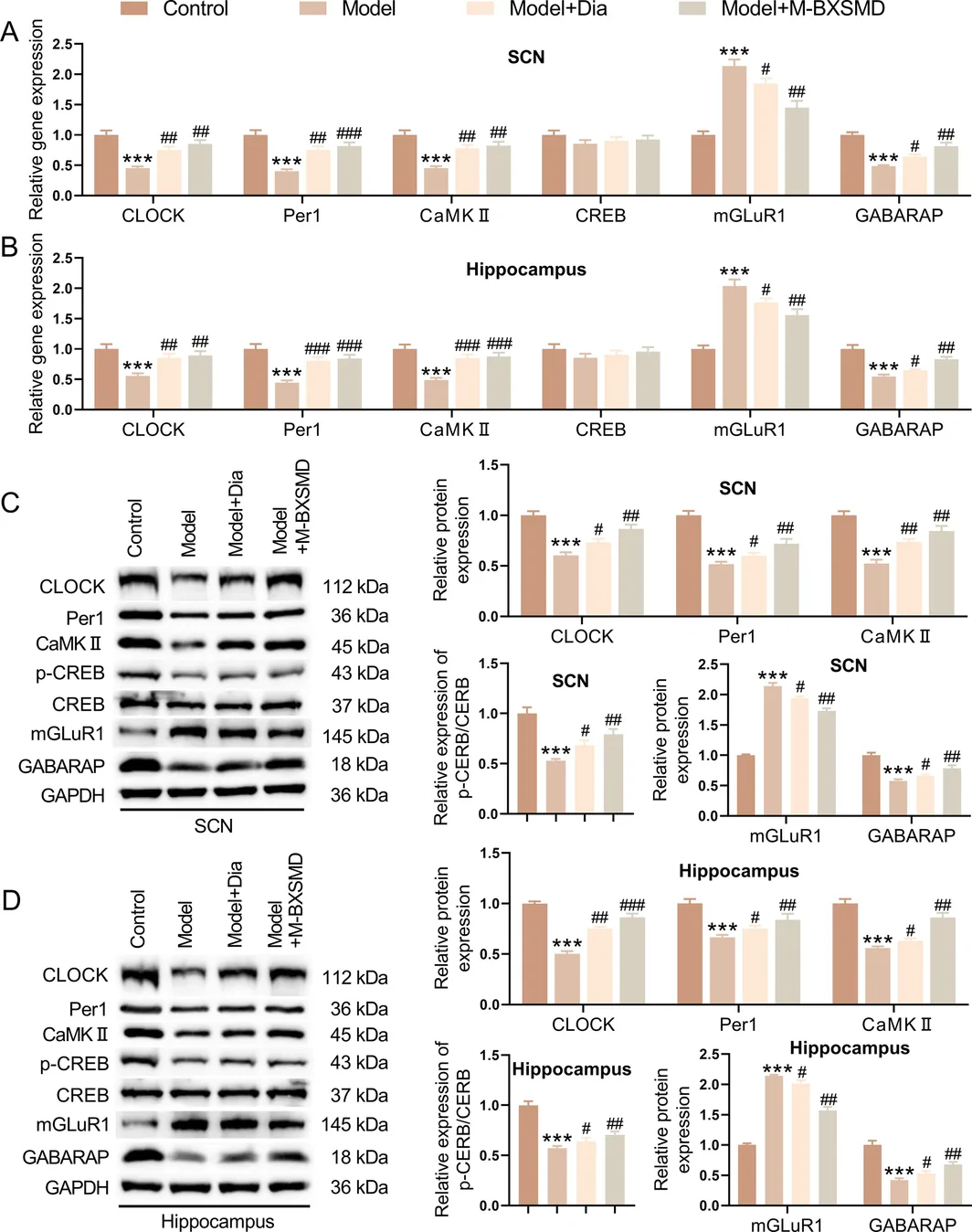
Effects of BXSMD on MT receptor‐related factors in insomnia rats. (A, B) qRT‐PCR measured the CLOCK, Per1, CaMKII, CREB, mGLuR1, and GABARAP gene expression levels in the SCN and hippocampus (A: F_(CLOCK)_ = 49.47, *p* < 0.0001; F_(Per1)_ = 54.82, *p* < 0.0001; F_(CaMKII)_ = 45.73, *p* < 0.0001; F_(CREB)_ = 2.660, *p* = 0.1194; F_(mGLuR1)_ = 81.92, *p* < 0.0001; F_(GABARAP)_ = 82.37, *p* < 0.0001; B: F_(CLOCK)_ = 26.37, *p* = 0.0002; F_(Per1)_ = 41.67, *p* < 0.0001; F_(CaMKII)_ = 40.97, *p* < 0.0001; F_(CREB)_ = 2.479, *p* = 0.1354; F_(mGLuR1)_ = 79.31, *p* < 0.0001; F_(GABARAP)_ = 69.79, *p* < 0.0001). (C, D) Western blot detected the expression of CLOCK, Per1, CaMKII, CREB, p‐CREB, mGLuR1 and GABARAP in the SCN and hippocampus (C: F_(CLOCK)_ = 61.01, *p* < 0.0001; F_(Per1)_ = 91.19, *p* < 0.0001; F_(CaMKII)_ = 72.07, *p* < 0.0001; F_(p‐CREB/CREB)_ = 53.50, *p* < 0.0001; F_(mGLuR1)_ = 518.2, *p* < 0.0001; F_(GABARAP)_ = 73.85, *p* < 0.0001; D: F_(CLOCK)_ = 203.9, *p* < 0.0001; F_(Per1)_ = 35.81, *p* < 0.0001; F_(CaMKII)_ = 101.3, *p* < 0.0001; F_(p‐CREB/CREB)_ = 94.5, *p* < 0.0001; F_(mGLuR1)_ = 424.9, *p* < 0.0001; F_(GABARAP)_ = 80.61, *p* < 0.0001). ***p < 0.001 vs. Control; ^#^ *p* < 0.05, ^##^ *p* < 0.01, ^###^ *p* < 0.001 vs. Model.

Previous studies have shown that MT decreased the expression of clock genes CLOCK and PER1 by interaction with MT1 [28]. MT also could up‐regulate CaMKII and p‐CREB levels via melatonin MT2 receptor [12]. GABA and Glu are crucial neurotransmitters in the central nervous system, capable of inhibiting and exciting neurons respectively, playing a decisive role in regulating sleep [29]. GABARAP is a key molecule regulating the efficacy of GABA [30]. At the same time, mGLuR1 is a key metabolic receptor that negatively regulates Glu, jointly maintaining the homeostasis and plasticity of neural circuits [31]. Thus, we tested the effects of BXSMD on MT receptor‐related factors. In the SCN and hippocampus, mRNA expression levels of CLOCK, Per1, CaMKII, and GABARAP were significantly decreased in the model group, while they were markedly increased in the Dia and M‐BXSMD groups. The mRNA expression level of mGLuR1 was significantly elevated in the model group, while Dia and the M‐BXSMD treatment significantly reduced the expression (Figure 4A,B). Compared to the model group, BXSMD significantly increased CLOCK, Per1, CaMKII, and GABARAP protein levels while decreasing mGLuR1 protein levels (Figure 4C,D). Notably, CREB mRNA expression showed no significant difference among groups (Figure 4A,B), while BXSMD significantly increased the p‐CREB/CREB protein ratio in both the SCN and hippocampus (Figure 4C,D). These results indicate that BXSMD modulates CREB activity through post‐translational phosphorylation rather than transcriptional regulation. Overall, these findings indicate that BXSMD regulates the expression of CLOCK, Per1, CaMKII, CREB, mGLuR1, and GABARAP in insomniac rats.

### 3.5 MT Receptor Antagonist Luzindole Reversed the Effects of BXSMD in Insomniac Rats

**FIGURE 5:**
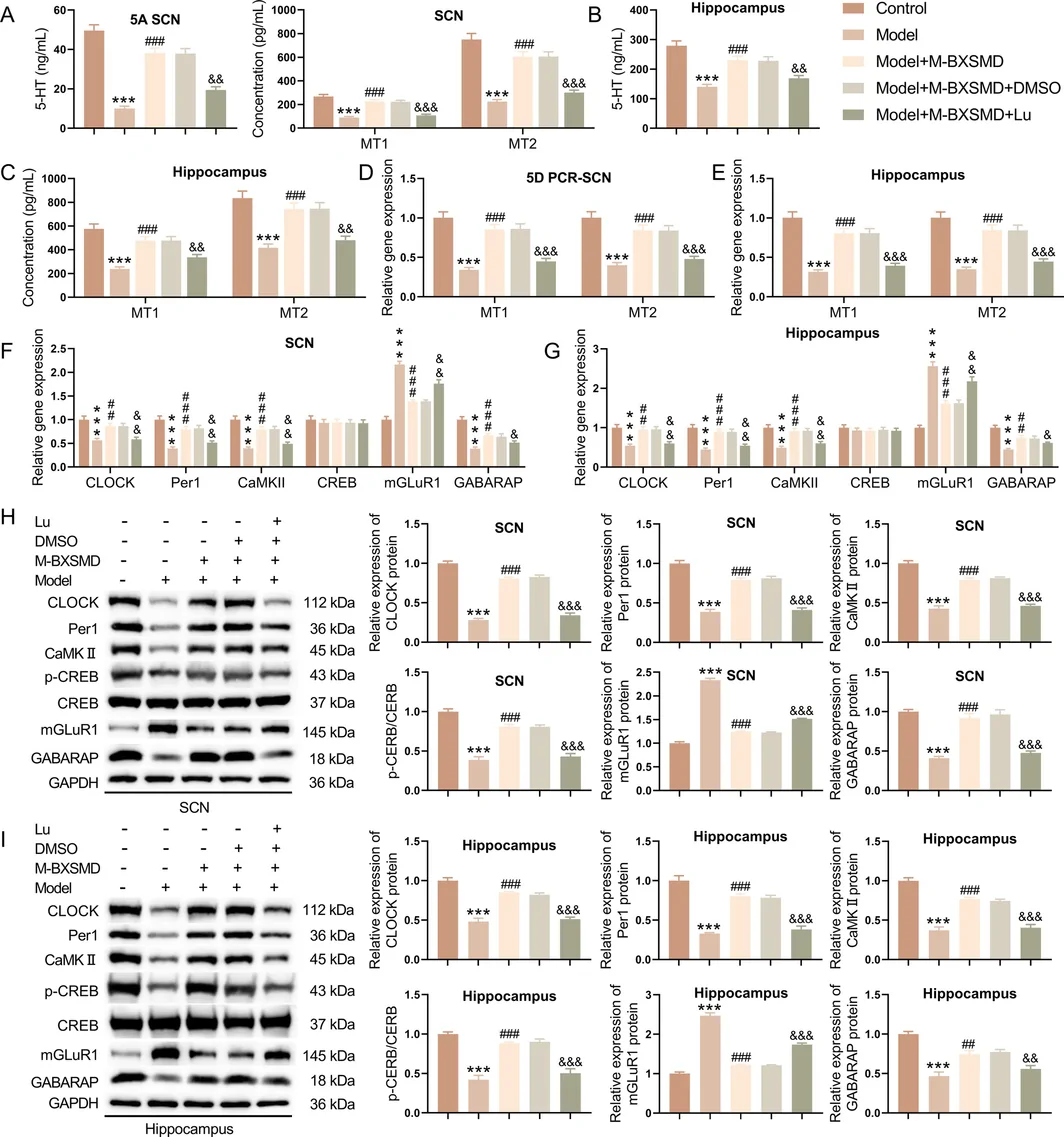
The MT receptor antagonist luzindole reversed the ameliorative effects of BXSMD in insomniac rats. (A–C) ELISA detected the 5‐HT, MT1 and MT2 content in the SCN and hippocampus (A: F_(5‐HT)_ = 151.5, *p* < 0.0001; F_(MT1)_ = 108.2, *p* < 0.0001; F_(MT2)_ = 112.2, *p* < 0.0001; B‐C: F_(5‐HT)_ = 56.54, *p* < 0.0001; F_(MT1)_ = 54.11, *p* < 0.0001; F_(MT2)_ = 44.93, *p* < 0.0001). (D, E) qRT‐PCR detection of MT1 and MT2 expression in the SCN and the hippocampus (D: F_(MT1)_ = 79.18, *p* < 0.0001; F_(MT2)_ = 63.45, *p* < 0.0001; E: F_(MT1)_ = 93.47, *p* < 0.0001; F_(MT2)_ = 75.41, *p* < 0.0001). (F, G) qRT‐PCR detection of CLOCK, Per1, CaMKII, CREB, mGLuR1, and GABARAP expression in the SCN and hippocampus (F: F_(CLOCK)_ = 32.86, *p* < 0.0001; F_(Per1)_ = 57.21, *p* < 0.0001; F_(CaMKII)_ = 65.23, *p* < 0.0001; F_(CREB)_ = 0.6045, *p* = 0.6683; F_(mGLuR1)_ = 162.7, *p* < 0.0001; F_(GABARAP)_ = 68.42, *p* < 0.0001; G: F_(CLOCK)_ = 36.59, *p* < 0.0001; F_(Per1)_ = 50.74, *p* < 0.0001; F_(CaMKII)_ = 44.31, *p* < 0.0001; F_(CREB)_ = 0.8056, *p* = 0.5489; F_(mGLuR1)_ = 130.3, *p* < 0.0001; F_(GABARAP)_ = 48.51, *p* < 0.0001). (H, I) Western blot detection of protein expression of CLOCK, Per1, CaMKII, CREB, p‐CREB, mGLuR1, and GABARAP in the SCN and hippocampus (H: F_(CLOCK)_ = 493.0, *p* < 0.0001; F_(Per1)_ = 245.7, *p* < 0.0001; F_(CaMKII)_ = 246.9, *p* < 0.0001; F_(p‐CREB/CREB)_ = 188.8, *p* < 0.0001; F_(mGLuR1)_ = 1354, *p* < 0.0001; F_(GABARAP)_ = 145.6, *p* < 0.0001; I: F_(CLOCK)_ = 159.5, *p* < 0.0001; F_(Per1)_ = 166.6, *p* < 0.0001; F_(CaMKII)_ = 179.5, *p* < 0.0001; F_(p‐CREB/CREB)_ = 122.3, *p* < 0 0.0001; F_(mGLuR1)_ = 557.2, *p* < 0.0001; F_(GABARAP)_ = 75.85, *p* < 0.0001). ***p < 0.001 vs. Control; ^##^ *p* < 0.01, ^###^ *p* < 0.001 vs. Model; ^&^ *p* < 0.05, ^&&^ *p* < 0.01, ^&&&^ *p* < 0.001 vs. Model + M‐BXSMD + DMSO.

To further validate that BXSMD acts through melatonin receptors, we utilized the MT receptor antagonist luzindole to perform antagonism experiments. Luzindole, a nonselective MT1 and MT2 antagonist, competitively blocks MT1 and MT2, inhibiting the effect of MT [32]. The results of ELISA showed that BXSMD increased the 5‐HT, MT1, and MT2 content in the SCN and hippocampus. However, this effect was partially reversed by the MT receptor inhibitor luzindole (Figure 5A–C). Subsequently, we investigated whether luzindole could reverse the expression of MT1, MT2, and MT downstream effector proteins. As indicated in Figure 5D–G, BXSMD significantly increased the mRNA expression of MT1, MT2, CLOCK, Per1, CaMKII, and GABARAP, while suppressing the expression of mGLuR1. Notably, CREB mRNA expression showed no significant difference among all groups. Luzindole partially reversed the regulatory effects of BXSMD on the mRNA expression of CLOCK, Per1, CaMKII, mGLuR1, and GABARAP, confirming that these effects are mediated through MT1/MT2 receptors. We also detected the protein levels of these molecules. BXSMD significantly upregulated CLOCK, PER1, CaMKII, p‐CREB/CREB, and GABARAP protein expression while downregulating mGLuR1 protein expression, and these regulatory effects were partially reversed by luzindole (Figure 5H,I). Collectively, these findings demonstrated that the MT receptor antagonist luzindole partially inhibited the regulatory effects of BXSMD on its downstream molecules in insomniac rats.

### 3.6 Luzindole Partially Reverses the Upregulation of ADAM10 Induced by BXSMD

**FIGURE 6:**
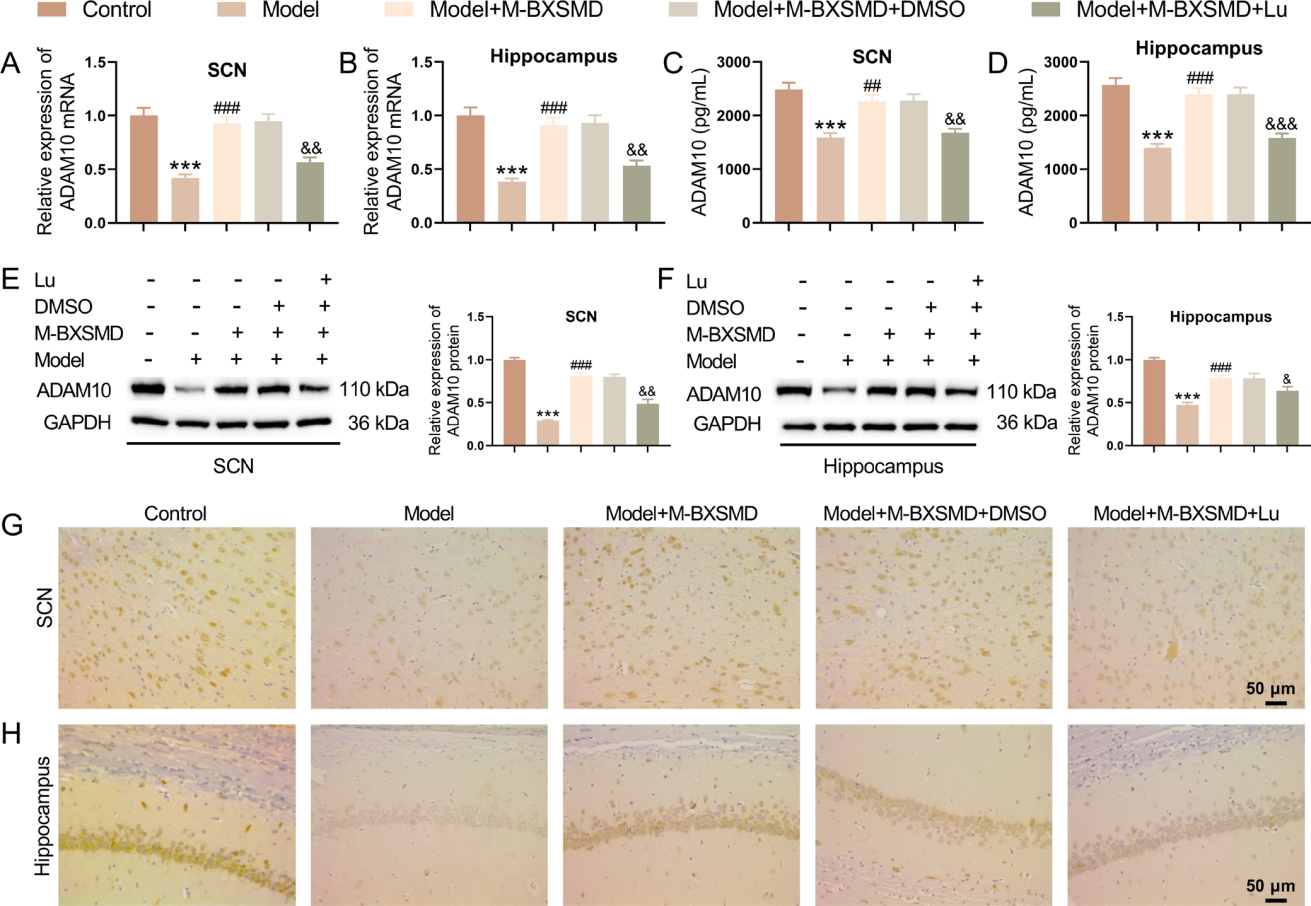
BXSMD regulates the expression of ADAM10 via MT receptor. (A, B) qRT‐PCR detection of ADAM10 gene expression in the SCN and hippocampus (F _(A)_ = 61.13, *p* < 0.0001; F _(B)_ = 61.23, *p* < 0.0001). (C, D) ELISA was used to detect the ADAM10 content in the SCN and hippocampus (F _(C)_ = 44.26, *p* < 0.0001; (F _(D)_ = 72.65, *p* < 0.0001)). (E, F) Western blot examined the expression of ADAM10 in the SCN and hippocampus (F _(E)_ = 217.8, *p* < 0.0001; (F _(F)_ = 70.79, *p* < 0.0001)). (G, H) IHC detected the ADAM10 protein level in the SCN and hippocampus. ***p < 0.001 vs. Control; ^##^ *p* < 0.01, ^###^ *p* < 0.001 vs. Model; ^&^ *p* < 0.05, ^&&^ *p* < 0.01, ^&&&^ *p* < 0.001 vs. Model + M‐BXSMD + DMSO.

MT can regulate the activity of ADAM10 protein through MT1 and MT2 receptors [19]. Can BXSMD regulate ADAM10 expression through MT receptors? We injected luzindole into insomniac rats to examine the effect of luzindole on ADAM10 expression. qRT‐PCR and ELISA assays detected ADAM10 expression level in SCN and hippocampus. Results showed that ADAM10 expression was significantly elevated in the M‐BXSMD group, while luzindole significantly inhibited this increase (Figure 6A–D). This phenomenon was observed in both Western blot and IHC experiments (Figure 6E–H). Therefore, luzindole partially inhibited the upregulation of ADAM10 induced by BXSMD, confirming that BXSMD modulates ADAM10 expression in a MT1/MT2‐dependent manner.

## 4 Discussion

Insomnia, one of the most important manifestations of sleep disorders, is highly prevalent in clinical practice, with a prevalence rate as high as 50% among primary care patients [33]. In this study, we investigated the therapeutic effects of BXSMD on a PCPA‐induced insomnia rat model. Behavioral assessments, along with evaluations of MT‐related indicators and downstream protein expression, were conducted to explore the efficacy and underlying mechanisms of BXSMD in treating insomnia.

Consistent with previous studies, PCPA exposure caused obvious insomnia‐like behaviors and learning and memory impairments in rats, and BXSMD treatment effectively ameliorated these behavioral abnormalities with obvious sedative‐hypnotic effects. In this study, we systematically confirmed that BXSMD synchronously modulates circadian molecules (CLOCK, PER1), CaMKII‐p‐CREB signaling, and synaptic homeostasis‐related molecules (GABARAP, mGluR1) in an MT1/MT2‐dependent manner. Most notably, we revealed that BXSMD upregulates ADAM10 expression through MT receptors in an insomnia model. Collectively, these findings demonstrate the therapeutic potential of BXSMD against PCPA‐induced insomnia and clarify its unique mechanistic characteristics. MT was found to modulate PCPA‐induced sleep/wake rhythms in insomniac rats and improve insomnia through its action on melatonin receptors in the SCN, and acts as an endogenous factor that regulates the circadian cycle [34]. MT regulates sleep by interacting with MT1 and MT2. MT1 regulates sleep mainly by mediating neuronal inhibition, and MT2 regulates circadian rhythms mainly by mediating sleep phase shifts [35]. The hippocampus and hypothalamic regions are considered to be key target areas in the pathogenesis of insomnia [36, 37]. According to research reports, many neurotransmitters in the brain are closely related to the occurrence and development of insomnia [38]. 5‐HT is a neurotransmitter in the brain that plays an important role in regulating sleep–wake rhythms and central fatigue. Our further studies confirmed that BXSMD treatment effectively increased levels of 5‐HT, MT, and their receptors MT1 and MT2 in key brain regions and peripheral tissues of insomnia‐induced rats. Increasing evidence suggests that traditional Chinese medicine can exert therapeutic effects on insomnia through the MT system. For example, Shen Yuan extract demonstrates sedative and hypnotic effects by upregulating levels of 5‐HT, MT, and their receptors (MT1/MT2) in the brain. Similarly, compound formulations such as Modified Suanzaoren Decoction [39] and Guhun Yangshengjing [40] enhance MT signaling in animal models of insomnia, representing a common key mechanism underlying the efficacy of TCM treatments for insomnia.

Oscillatory rhythms expressed by Per1 and CLOCK, two of the most important biological clock genes/proteins involved in the transcription‐translation loop and controlling core circadian rhythms, have been reported to be severely disrupted upon MT1 knockdown [41]. cAMP response element binding protein (CREB), an intranuclear regulator with neuroprotective effects, has been implicated in insomnia [42]. CaMKII may be involved in insomnia by participating in the calmodulin signaling pathway [43]. Recent studies have demonstrated the role of CaMKII phosphorylation status in promoting sleep through AAV‐mediated expression of CaMKIIβ and its phosphorylation‐mimetic mutant [44]. There is evidence that MT2 activated by MT promotes an increase in intracellular calcium ions as well as CaMKII expression and increases CREB phosphorylation levels [45]. In our study, BXSMD promoted the gene and protein expression levels of CLOCK, Per1, CaMKII, and p‐CREB in PCPA‐induced insomnia rats. This effect was partially reversed following intervention with the MT inhibitor luzindole, indicating that BXSMD enhances the expression of CLOCK, Per1, CaMKII, and p‐CREB by activating the MT signaling pathway.

GABA and Glu are important neurotransmitters in the central nervous system that inhibit and excite neurons, respectively, and play a decisive role in the regulation of sleep [46, 47]. GABAergic enhancement depends on the GABARAP scaffold protein. GABARAP helps maintain the brain's “excitatory‐inhibitory” balance by enhancing the efficacy of the endogenous GABAergic inhibitory system. Its normal function serves as a crucial molecular basis for promoting natural sleep [48]. Previous studies combined with thalamocortical (TC) restricted‐PLCβ4 knockdown data demonstrated that the corticothalamic input to TC neurons through the mGLuR1‐PLCβ4 pathway was critical for sleep architecture and the generation of sleep rhythms [44, 49]. In this text, the insomnic rats exhibited a decrease of GABARAP and the increase of mGLuR1 gene and protein. Compared with the model group, BXSMD up‐regulates the expression of GABARAP and downregulates mGLuR1 gene and protein in the hippocampus and the SCN of the hypothalamus of rats. Importantly, these regulatory effects of BXSMD on GABARAP and mGluR1 were partially reversed by co‐administration of the MT receptor antagonist luzindole, indicating that BXSMD modulates the GABARAP/mGLuR1 balance in a MT receptor‐dependent manner. This finding further supports that BXSMD restores excitatory‐inhibitory homeostasis through MT1/MT2‐mediated signaling, thereby contributing to its sleep‐promoting effects.

ADAM10 is a type I transmembrane protein composed of approximately 750 amino acids, which has been widely recognized as an α‐secretase based on extensive in vitro and in vivo evidence [19]. When MT receptors MT1 and MT2 on the plasma membrane are stimulated, the Gq/PLC/PKC, Gi/PI3K/PDK1/PKC, and Gs/cAMP/PKA signaling pathways are activated, leading to the phosphorylation of ERK1. ERK1 can phosphorylate CREB and Oct‐1, thereby causing increased transcription of ADAM10 [20]. In our study, ADAM10 expression was suppressed in insomnia‐prone rats, while BXSMD promoted ADAM10 expression by activating the MT signaling pathway. Notably, although ADAM10 has been extensively studied in the context of Alzheimer's disease, cancer, and inflammation [50], its role in insomnia has remained largely unexplored. This study is the first to demonstrate that BXSMD upregulates ADAM10 expression in an insomnia model, and that this upregulation is partially mediated through MT1/MT2 receptors, as evidenced by the partial reversal effect of luzindole. The consistent partial reversal by luzindole across all outcomes indicates that while MT receptor activation is a key mediator of BXSMD's anti‐insomnia effects, it is not the sole pathway; additional MT‐independent mechanisms may also contribute, and further investigation into these mechanisms is warranted. These findings not only expand the functional landscape of ADAM10 beyond its canonical roles in proteolytic shedding and neurodegeneration, but also identify ADAM10 as a downstream effector in the MT signaling axis underlying insomnia pathophysiology. Thus, ADAM10 may represent a novel molecular node for future therapeutic intervention in sleep disorders.

In summary, this study confirms that BXSMD effectively alleviates PCPA‐induced insomnia‐like behaviors in rats. Mechanistically, BXSMD modulates the expression of sleep‐related molecules (CLOCK, PER1, CaMKII, p‐CREB/CREB, GABARAP, mGluR1) and elevates ADAM10 expression through MT1/MT2 receptor‐dependent signaling. This finding reveals the multi‐targeted, multi‐level action characteristics of BXSMD and elucidates its central functional coupling mechanism mediated by MT receptors. Future research should further elucidate the core active components targeting the MT‐ADAM10 axis in BXSMD, clarifying more specific regulatory mechanisms to provide a more robust experimental basis for the modernization and clinical application of this classical formula.

## 5 Conclusion

This study demonstrated that BXSMD effectively alleviated insomnia‐like behaviors in PCPA‐induced rats. BXSMD increased the levels of 5‐HT, MT, and MT1/MT2 receptors, and modulated the expression of sleep‐related molecules (CLOCK, PER1, CaMKII, p‐CREB/CREB, GABARAP, mGluR1), as well as ADAM10. Mechanistically, blockade of MT1/MT2 with luzindole partially reversed the above regulatory effects, indicating that BXSMD modulates the expression of these sleep‐related molecules and ADAM10 in an MT1/MT2‐dependent manner in PCPA‐induced insomniac rats.

## Author Contributions

**Baojun Guo:** conceptualization, data curation, formal analysis, investigation, writing – original draft, writing – review and editing. **Yuqin Tang:** conceptualization, data curation, formal analysis, writing – original draft, writing – review and editing. **Yongqiang Zhang:** software, methodology, writing – review and editing. **Yeke Wu:** project administration, resources, writing – review and editing. **Ranran Gao:** conceptualization, writing – review and editing.

## Funding

This study was supported by the Postdoctoral Research Project in Henan Province (335953), the National Natural Science Foundation of China (81973684), the Natural Science Foundation of Sichuan Province (2023NSFSC1760), the Henan Province Postdoctoral Research Program (HN2024079), the Health Commission of Chengdu, and the Chengdu University of Traditional Chinese Medicine Joint Innovation Fund in 2024 (WXLH202402019).

## Ethics Statement

All animal experiments were conducted with the approval of the Experimental Animal Ethics Committee of Henan Provincial Academy of Traditional Chinese Medicine (HNTCMDW‐2023092301).

## Conflicts of Interest

The authors declare no conflicts of interest.

## Data Availability

The data that support the findings of this study are available from the corresponding author upon reasonable request.
